# A DSL-Based Approach for Detecting Activities of Daily Living by Means of the AGGIR Variables

**DOI:** 10.3390/s21165674

**Published:** 2021-08-23

**Authors:** José Manuel Negrete Ramírez, Philippe Roose, Marc Dalmau, Yudith Cardinale, Edgar Silva

**Affiliations:** 1 LIFO, Institut National des Sciences Appliquées Centre Val de Loire, Université d’Orléans, 18000 Bourges, France; 2 IUT de Bayonne-LIUPPA, Université de Pau et des Pays de l’Adour, 64600 Anglet, France; philippe.roose@iutbayonne.univ-pau.fr (P.R.); Marc.Dalmau@iutbayonne.univ-pau.fr (M.D.); 3 Departamento de Computación y Tecnología de la Información, Universidad Simón Bolívar, 1080 Caracas, Venezuela; ycardinale@usb.ve (Y.C.); 11-10968@usb.ve (E.S.)

**Keywords:** domain specific language, feature-oriented programming, pervasive computing, pervasive health systems and services, AGGIR grid

## Abstract

In this paper, we propose a framework for studying the AGGIR (Autonomie Gérontologique et Groupe Iso Ressources—Autonomy Gerontology Iso-Resources Groups) grid model, with the aim of assessing the level of independence of elderly people in accordance with their capabilities of performing daily activities as well as interacting with their environments. In order to model the Activities of Daily Living (ADL), we extend a previously proposed Domain Specific Language (DSL), by defining new operators to deal with constraints related to time and location of activities and event recognition. The proposed framework aims at providing an analysis tool regarding the performance of elderly/disabled people within a home environment by means of data recovered from sensors using a smart-home simulator environment. We perform an evaluation of our framework in several scenarios, considering five of the AGGIR variables (i.e., feeding, dressing, toileting, elimination, and transfers) as well as health-care devices for tracking the occurrence of elderly activities. The results demonstrate the accuracy of the proposed framework for managing the tracked records correctly and, thus, generate the appropriate event information related to the ADL.

## 1. Introduction

According the United Nations Department of Economic and Social Affairs (UN DESA) (https://www.un.org/development/desa/publications/world-population-prospects-the-2017-revision.html, accessed on 18 July 2021), the population is aging faster than ever before. Due to this increase, reducing medical costs and improving quality of care service [1] have become, in recent years, requirements for new care delivery mechanisms and structures [2]. Personal Sensor Networks (PSN) and Body Sensor Networks (BSN) in smart environments have become viable alternatives to traditional healthcare solutions. PSNs are used to detect human daily activities and measure conditions within the environment. BSNs are used to monitor vital signs and health conditions by measuring physiological parameters.

Several approaches propose different frameworks by focusing on identifying the Activities of Daily Living (ADL) that require monitoring [3,4,5,6,7,8,9,10,11,12]. However, there is still a lack of ADL to be analysed, whereas others consider a subset of specific activities. However, most importantly, they are not based on a specific tool such as the AGGIR (Autonomie Gérontologique et Groupe Iso Ressources—Autonomy Gerontology Iso-Resources Groups) grid [13]. The AGGIR grid is an autonomy assessment tool used in France for measuring the independency level of elderly people.

In this context, in a previous work [14], we presented a DSL that allows the representation of atomic activities composing the AGGIR grid in the form of a plot, providing a history file for detecting abnormal behavior of the inhabitants in the monitored house. Afterwards, we developed a framework in which the DSL is integrated [15], with the aim to achieve the monitoring of a person within a smart home environment to identify the ADL performed by the inhabitants by means of collection and analysis of data obtained from sensors located in the environment over a certain period of time.

In this paper, we extend our previous works by introducing the identification of complex activities and providing support for simulation and visualization. In order perform this, the DSL is extended by defining operators that deal with constraints related to time, location, and event recognition. Moreover, this DSL is able to categorise a set of activities based on the constants of the AGGIR grid, which classifies autonomy levels to various environmental factors affecting the activities and social life of a person. Thus, more AGGIR activities can be detected, such as alimentation, dressing, toileting, elimination, and transfers. The framework is improved by adding support for more types of sensors and the capacity of performing configuration on the environments and visual simulations of the behaviour of people in such environments. Thus, the framework provides an analysis tool regarding the performance of elderly/disabled people within a home environment by means of data recovered from sensors using the iCASA simulator. In turn, the framework provides a general approach to detect ADL, relate them with the AGGIR variables, and to determine the independence of elder people in their homes. In order to evaluate our approach, we pick five of the AGGIR variables (i.e., feeding, dressing, toileting, elimination, and transfers) and evaluate their testability in many scenarios by means of records representing the occurrence of activities or unexpected behavior of the elderly. Furthermore, a health-care device use case concerning the employment of medical equipment for tracking blood glucose is presented. The results demonstrate the accuracy of our framework in managing the obtained records correctly and, thus, generating the appropriate event information.

In summary, our main contribution in this work is a framework aimed at providing an evaluation tool based on the AGGIR model regarding the performance of ADL by elderly/disabled people within a home environment by processing data recovered from sensors.

The remainder of this paper is organized as follows. The AGGIR grid model as well as the definition and characteristics regarding different types of DSL are explained in Section 2. Related studies are presented in Section 3. The main features of the proposed DSL, as well as a brief review of operators regarding time, location, and event constraints, is described in Section 4. Our framework proposal is explained in Section 5. The details of the experiments and the discussion about obtained results are provided in Section 6. Finally, conclusions and future work are provided in Section 7.

## 2. The AGGIR Grid Model and DSL

In this section, the AGGIR grid model, a tool introduced in January 2002 for expanding senior benefits, is briefly described. Additionally, a review of the different types of DSL in any context (logic languages, textual languages, and graphical languages) is presented.

### 2.1. AGGIR Model

The AGGIR grid is an autonomy assessment tool used in France to measure the independency levels of elderly people. The AGGIR grid model is a six-level dependence scale (GIR1 to GIR6) that can be defined based on a set of seventeen three-state variables. Each variable can have one of these values: (A) for complete dependency; (B) for partial dependency; and (C) for complete independency. The variables are classified into the following two groups: discriminatory and illustrative variables, as shown in Table 1.

Since DSLs are recognized as effective tools for increasing the productivity and quality of software development [16,17,18], in this work we propose a DSL in order to express the situations related to the AGGIR variables that respond to the ADL performed by people with physical or mental disability, support for elderly, and diseases connected to aging. The DSL allows expressing situations related to the maintenance of people at home [19]. Subsequently, the definition and characteristics regarding several DSL categories are presented.

### 2.2. Domain Specific Languages (DSL)

A DSL is a programming language that helps developers in defining concepts in terminology belonging to a particular domain [20,21,22,23].

DSLs are conceived as small textual languages that are able to take certain narrow parts of programming and make them “easier to understand and therefore quicker to write, quicker to modify, and less likely to breed bugs”. Their main goal is to improve developers’ productivity as well as reducing the communication gap in software development between programmers and domain experts [24]. Moreover, DSLs are considered increasingly popular software development techniques, that use concepts from the problem domain rather than the solution domain [25].

DSLs are separated into two categories based on their design: internal DSL and external DSL [24]. An internal DSL (also called embedded DSL) provides a general-purpose host language to solve domain-specific problems (originally based on the LISP programming language) [26]. Due to the fact that an external DSL is independent from any other language, they require their own infrastructures such as parsers, linkers, compilers, or interpreters [27]. External DSL represent 50% of DSL [28].

Moreover, patterns [20] and design guidelines [29] have been proposed for the development of DSL. These guidelines are classified by the following: purpose, realization, content, concrete, and abstract syntax, as shown in Table 2. According to the concrete language, purpose, and domain, some guidelines might be contradicting or irrelevant [29]; thus, they should be considered as proposals when designing a DSL [30]. The most common approach is to write requirements using natural language [31]. This can be used for different purposes to describe any kind of requirements and functional specifications for information systems [32].

## 3. State of the Art

Several DSLs have been developed for different areas of application, e.g., expert rules, business rules, and configuration rules. A systematic mapping study is presented in [28].

The conceptual DSL described in [33] expresses how business logic can be translated by means of automated code execution for the creation of logic-based smart contracts. Moreover, two execution modes for smart contracts are highlighted: lazy execution (initiated by an actor, either manually or scheduled) and eager execution (fully automated transactions).

IoTDSL, a prototype DSL meant to allow end users to drive the IoT devices installed in their homes relying on a high-level rule-based language is presented in [34] in order to achieve definition and manipulation of devices that are deployed in home environments. Users are able to describe and combine event-based semantics as well as structural configurations in a declarative manner, with high-level representations of devices. The orchestration of events is then analysed to a component in charge of translating high-level rules into a complex event processing facility dedicated to evaluating runtime events. Moreover, the proposed prototype relies upon textual syntax, and the simulation code can be generated to play configurations defined by the user.

A framework is proposed in [35] that uses logical and probabilistic reasoning approaches with respect to complex event reasoning concerning process, integration, and provision of reasoning in order to address decision support relative to supply chain planners whenever information about disruption events (such as process failure) is incomplete or uncertain.

A DSL called ReqDL for describing requirements and capturing bidirectional traceability data concerning system modeling elements is introduced in [31]. Additionally, a generation algorithm for independent trace models is presented, as well as concrete and abstract syntax in terms of grammar and metamodel. Moreover, three types of operators are proposed for describing requirements and for capturing trace data: attributes, description, model elements, and trace links. Moreover, a working example is also provided.

A graphical DSL provides domain experts with an intuitive and user-friendly tool for defining the complex event processing domains of interest for which critical situations in real time need to be detected [36]. Some recent works on this research field are presented.

Dolphin, an extensible programming language implemented as a Groovy DSL for autonomous vehicle networks, is described in [37]. It is designed to express an orchestrated execution of tasks defined for multiple vehicles that are dynamically available in a network. Additionally, the built-in operators include support for composing tasks in several forms, i.e., instance according to concurrent, sequential, or event-based task flow, partially inspired by process calculi approaches. Moreover, the integration of the aforementioned DSL with an open-source toolchain for autonomous vehicles is described in which users are able to edit and run Dolphin programs by using a custom window embedded in the overall GUI environment for editing and monitoring. Furthermore, the results from field tests using unmanned underwater vehicles and unmanned aerial vehicles are presented.

The authors of [38] introduced a DSL named SimulateIoT, a model-driven development approach aiming to define, generate code, and deploy the simulation of IoT systems, thus achieving their design as well as their deployment by means of a domain metamodel, a graphical concrete syntax, and a model to text transformation.

A DSL called ALPH for ubiquitous healthcare is presented in [39]. It aims to cover the following: (i) mobility, by assisting users concerning devices disconnections; (ii) context-awareness regarding the adaptation to environmental changes of the application behaviour; and (iii) infrastructure, aiming to control the communication protocol heterogeneity.

In order to assist pathway-supporting health information systems, the authors of [40,41] proposed a domain specific modeling language with the objective of developing clinical pathways by taking into consideration the conception, modeling, realization, and impact of the above-mentioned subject.

All these works demonstrate the utility of different types of DSL in several domains. With the purpose to support the identification of ADL performed at home by elderly/disabled people, many works have proposed the development of DSL. In the following section, we dedicate the discussion on the revision of existing DSL in the context of both description and recognition of ADL. Furthermore, a brief review of operators regarding time, location, and event constraints is introduced.

### 3.1. DSL for the Detection of ADL

The authors of [42] introduced a smart city oriented infrastructure for collecting and managing data related to behavior patterns concerning elderly people and their daily activities in both indoor and outdoor environments. Validation considering both low-level and high-level use cases was carried out. Part of the grammar employed regarding user motility and indoor-outdoor localization is presented: *BODY_STATE_START/ BODY_STATE_STOP* for indicating the change of a particular body state of the subject (i.e., still, walking, sleeping, etc.); and *POI_ENTER/POI_EXIT* for managing the location type and/or the GPS coordinates. Furthermore, the user-environment interaction is described by activities such as *FURNITURE_OPEN/ CLOSED* for sensing contact, motion, and vibration on furniture and tools. Moreover, *state_type* and *location_type* are defined in order to identify the performance of activities.

The authors of [43] proposed a DSL for model-driven development of activity-oriented context-aware applications in order to facilitate development by improving the efficiency of developers. The concept model of such applications is analyzed in order to design abstract syntax and concrete syntax. The implemented tools include the development environment as well as the generation of Java code and ontology specification. A case study and evaluation concerning a smart meeting room was introduced in order to demonstrate the utilities of the proposed approach.

An ambient assisted living architecture for the monitoring of elderly people was presented in [44]. The system is able to collect all information coming from the heterogeneous sensors located in the indoor environment, such as environmental parameters or biomedical information, and to forward the information towards a remote service. The proposed system is able to continuously monitor elderly locomotor activity. Additionally, the system can trigger specific events when dangerous situations occur (e.g., fall detection). The feasibility of the proposed architecture was demonstrated by validation functional tests, implementing a real supporting tool for the elderly subject during his daily life activities.

The authors of [45] presented a study based on a model-driven solution for a top-down approach for rapid design and prototyping of ambient assisted living capable of detecting the behaviour of elderly persons in their home by acquiring data through a sensor tag wristband that sends data to a smartphone application through Bluetooth low energy protocol. The list of the detected low-level activities are as follows: body state, indoor home monitoring, presence in indoor places, presence in outdoor places, smartphone usage, usage of home appliances, interaction with transportation, and ambient parameters. Moreover, an example of grammar for low-level activities for the moving status concerning the *start* and *stop* moving actions is presented. It includes *START_MOVING* and *STOP_MOVING*. Furthermore, it is possible to calculate the duration of body state status along with the *STILL_TIME* value. Such a duration can be computed and treated as a measure.

A DSL for processing online events targeting binary sensors is presented in [46]. The approach is limited to handling binary sensors due to the fact that the proposed operators are defined on the domain of Boolean signals. The aforementioned language provides a primitive notion of state, modeled as a Boolean signal over time, and allows the generation of complex conditions on different states by using signal operators. Additionally, an interpreter for the language was implemented and applied in order to rewrite a set of real ambient assisted living services.

A tool-based methodology aimed to track the ADL of elder adults supporting replicable research is proposed in [47]; it permits the processing of sensor data with the purpose of defining a ruled-based monitoring process regarding the detection of the above-mentioned activities. Moreover, it is intended to assist professional caregivers by providing functional awareness by means of a graphical tool and by taking advantage of user specific information and abstracting both complex and typical situations.

An approach with the objective to evaluate a DSL focused on assistive services by professional caregivers is presented in [48]. Such an end-user language enables the formulation of assistive services by employing a domain-specific terminology. Moreover, the features concerning the representation of circumstances where assistance needs to be provisioned are introduced, in addition to identifying the necessary interventions to be considered if assistance is required within context-aware systems. The DSL is only dedicated to the detection of contexts.

The research in [49] addresses a DSL conceived to support users with disabilities throughout the performance of a semi-automated cooking process. Furthermore, a graphical user interface (GUI) is furnished in order to access a set of instructions located within a cloud-based repository regarding meal preparation, including microwave cooking directions. Additionally, it relies on a barcode scanner, a touchscreen, and a set of speakers, as well as a set of environmental sensors. Audio and visual alerts regarding safety concerns are also provided.

Table 3 summarizes and evaluates the most recent DSL approaches regarding the services they provide and the categories of monitored daily activities, sensing types, and geriatric models.

All these works consider the correct identification of the activities that require analysis of ADL. However, there is still a lack of ADL to be detected while others only focus on a subset of specific activities. However, most importantly, they are not based on a specific tool, such as the AGGIR grid.

### 3.2. Temporal, Localization, and Events Operators: A Review

In this section, we present a review of different operators used to describe time and location relations among events. Actions and activities that can be identified in a space are called events. These events can be tagged with time and location that, in turn, render events related according to the moment and location in which they occur.

According to the study presented in [50], in order for a system to make sensible decisions, it has to be aware of where the users are and have been during some period of time. Spatial and temporal logic is a well established area of Artifitial Intelligence (AI) [51], which has been applied to represent and reason on spatial and temporal features and constraints of context and situations [52]. For instances, temporal knowledge on human activities can be specified by means of the temporal operators ANDlater and ANDsim in Event-Condition-Action rules introduced in [53]. The authors of [54] applied Allen’s Temporal Logic [55] (see Table 4) to describe, constrain, and reason on temporal sequences in dealing with temporal and spatial knowledge in smart homes as well.

The event calculus of Shanahan [56], infers what is true from information that expresses what and when something occurs (actions) and what happens after those actions. Shanahan uses the action concept instead of event, but it makes no difference between the two. The notion of "fluent" is used to express anything that can change over time, as seen in Table 5.

Some others works worth revising are shown in Table 6, where temporal, spatial, and event-related operators are proposed in order to provide a significant contribution to a solution of these problems. We consider the temporal operators proposed by Allen [55], as well as operators such as “*Before, During, After, Begin, End, startTime, and endTime*”. With respect to location operators, we employ “*Inside, Outside, Joint, and nextTo*”, among others. We also utilise conventional mathematical operators such as “*greater than, lower than*”athematic operators to indicate event conditions based on attributes.

## 4. Domain Specific Language

In a previous work, we developed a DSL [14] in order to express situations related to the AGGIR variables that respond to the activities performed by people with physical or mental disability to determine their independence at home. Afterwards, we developed a framework [15] in which the DSL is integrated and intended to supervise an occupant within a smart home environment aiming to recognise several ADLs carried out by the household residents over a certain period of time. In this paper, we extend the DSL by introducing the identification of complex activities. In this context, an atomic event is defined as an event that can be detected by using one reading from a sensor; whereas a complex event is considered as an activity performed by an inhabitant that is detected by several sensors at the same time.

For carrying out such identification, the sensors for identifying the ADL are specified (Section 4.1 and Section 4.2), as well as the orchestration for atomic (Section 4.4.1) and complex events detection (Section 4.4.2). Moreover, the features that the extended DSL provides are the recovery of data from health related sensors and home-related sensors defined and described through the DSL in order to register operations regarding a specific date or a time range starting from a particular date or between two-time points, in addition to the identification of both atomic and complex events.

### 4.1. Smart Home Sensors

In the context of this work, the purpose of Wireless Sensor Networks (WSN) is to detect a user’s vital signs, activities, and surroundings. In order to achieve this goal, a set of sensors is needed to measure vital signs from the inhabitant. Moreover, smart home sensors need to be deployed to monitor the surroundings in a home environment; thus, it is possible to identify the activities associated with the AGGIR constants by considering physiological contexts and environmental contexts.

Thus, for developing the proposed DSL, it is necessary to acknowledge which sensors must be employed for this matter. With regard to the identification of ADL by means of several sensors for providing non intrusive monitoring, a group of sensors that can describe such activities are proposed. The aforementioned sensors are listed in Table 7, where some examples of their use are proposed. Additionally, data attributes and data types for each sensor are indicated.

Moreover, relevant data for each performed task identified through the data gathered by sensors are considered in Table 8 in order to obtain information to achieve the identification of the AGGIR constants.

### 4.2. Health Related Sensors

As previously mentioned, health care issues within the aging population represent a problem that needs to be addressed in the proposed DSL. Regarding this aim, a group of commercial medical sensors are suggested; such sensors are usually considered non-intrusive, replaceable, and most of them are low cost. Medical sensors are mainly used in the medical field for the objective of pervasive healthcare. Some of the conventional medical sensors for physiological measurements are listed in Table 9. Moreover, each of the devices has specific requirements in terms of parameters, units, and collected data.

### 4.3. Main Characteristics of the Proposed DSL

Due to the fact that we need to understand the process under which the proposed DSL works, it is relevant to highlight its most remarkable characteristics: (i) expressing situations related to the AGGIR variables that respond to the activities performed by the elderly/handicapped people at home; (ii) the representation of attribute-based event conditions; (iii) the representation of spatio-temporal event conditions. These are defined by temporal operators, such as *Before, During, and After*. To this end, the representation of the DSL relies on a GUI in order to describe complex events.

**DSL interface**. We propose a graphical DSL rather than textual language in order to make it easy to handle since the user might not necessarily be a person who is acknowledged in the programming field. Data recovery from sensors is crucial for the detection of ADL. For this matter, the proposed DSL plays an important role by representing the gathering of information related to the sensed environments, as well as returning graphical results by means of the aforementioned GUI. Additionally, the information present in the results can be saved as textual specifications in order to be reused and, thus, facilitates finding analysed data without performing any manipulation once again. This functionality allows its reusability by different context-aware applications and, thus, is not limited only to the AGGIR variables.

**DSL operations**. The textual specifications generated by DSL GUI can be used to describe events from an operation perspective, such as an operation in charge of measuring one of the following options: a value, a maximum or minimum value, an average of a set of values, or graphically displaying measured values within a time domain. These operations are exposed as a programming interface as follows: (i) the Value operation returns the value (*s*) of the device; (ii) the Maximum operation returns the maximum value of the value (*s*) of the device; (iii) the Minimum operation returns the minimum of the value (*s*) of the device; (iv) the Average operation returns the average of the value (*s*) of the device; and (v) the Graphic operation returns an icon with the value (*s*) of the device.

Due to the fact that events within a smart home environment are sensed during a specific time range, multiple possibilities for describing time using the DSL are considered. This includes determining a specific date or description of a time range starting from a particular date or between two-time points representing the beginning and end of the event. The specified time points can be either a date for a day or hours within a day. The Value operation can be calculated for the following values: (i) on a specific date; (ii) between two hours; (iii) between two dates; (iv) from a specific date. The operations Maximum, Minimum, Average, and Graphic are calculated for the following values: (i) between two dates; (ii) from a date; and the (iii) total (all available values for the device). After selecting the operation as well as the desired calculation means, it only remains to specify the date or the time according to what has been previously selected.

Since, in the proposed DSL, the need to represent activities performed by the elderly or handicapped people within a home environment is present, it is imperative to be aware of when such an activity takes place, as well as if there exists repetition or periodicity during the interval of its performance. By implementing such a methodology, the detection of anomalies on the behaviour of the inhabitants is intended; thus, the coherence of the ADL can be ensured.

**Spatio-temporal event conditions**. In order to deal with temporal constraints, spatio-temporal event conditions are also represented by the DSL. Those are defined by temporal operators such as “*Before, During, After, Begin, and End*” and are defined using spatial operators such as “*Inside, Outside, and Joint*”. The proposed criteria for determining the parameters for the example of the preceding paragraph include the following: time and space and within the time dimension. Moreover, it is relevant to consider the following:

(i) Concurrency: For recognizing activities that take place simultaneously, but they do not necessarily require the user’s interaction at the same time. That is, activities that have been started but not yet ended by the inhabitant [70].
(ii) Precedence: For establishing a logical order of the activities, e.g., going to the bathroom and then washing hands.
(iii) Simultaneity: For identifying the activity that takes the most amount of the time from the user when multitasking capabilities might be present, e.g., preparing meals and calling on the phone or watching television while eating.
(iv) Recurrence: For determining a logical sequences of situations. In the case where there is a recurrence of an activity, it is essential to define what this activity is and whether the activity iscarried out regularly.

**Figure 1:**
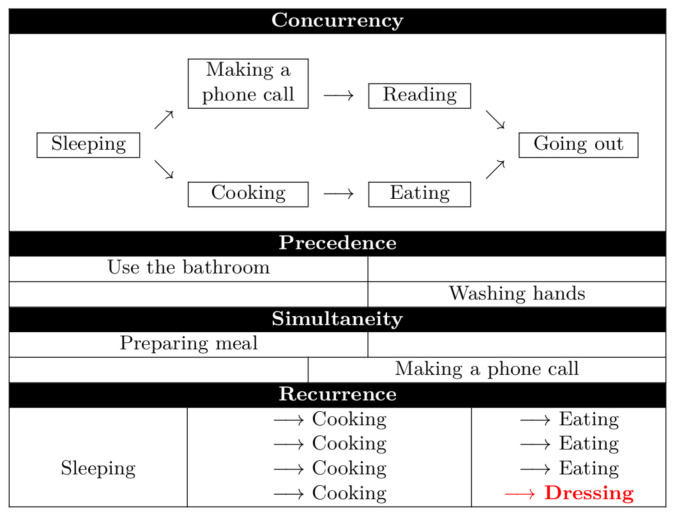
Criteria proposed over the time dimension.

Some examples of these four are presented in Figure 1.

### 4.4. Representation of the AGGIR Variables

So far, the main characteristics of the proposed DSL have been introduced. In this section, the explanation regarding the orchestration for both atomic and complex event detection is provided.

#### 4.4.1. Atomic Event Detection

In order to recognize an atomic event, an evaluation of a given sensor reading against a predefined condition from a simple activity is required. In the proposed approach, an atomic event is simply defined as an *Activity*, and each ***Activity*** represents one of different tasks composing every AGGIR variable. Table 10 shows how each ***Activity*** is associated with data obtained from sensors located within the smart-home environment concerning three of the AGGIR variables: toileting, dressing, and transfers. For the toileting AGGIR variable, three atomic events extracted from sensor readings are considered: the bathroom door sensor identifies the ***Activity*** regarding opening/closing the door; the toilet flush sensor indicates the ***Activity*** of activating the toilet flush; and the washbasin proximity sensor describes the ***Activity*** of washing hands.

#### 4.4.2. Complex Event Detection

Following the description of atomic event detection, it is important to emphasize how complex event detection takes place in the proposed approach. For this subject and parting from the readings from sensors defining a simple activity, a complex event can be considered as a sum of multiple events connected together representing each of the AGGIR grid variables. That is, an event that summarizes, represents, or denotes a set of atomic events (Activities). In the proposed approach, a complex event is stated clearly as a *Situation*.

In order to illustrate the composition of a *Situation*, Table 11 indicates how three of the AGGIR variables are composed. The (i) toileting *Situation* is composed of the following ***Activities***: *open bathroom door*, *use of toilet flush*, *wash hands*. The (ii) dressing ***Situation*** is composed of the following ***Activities***: *open wardrobe door*, *spend time changing clothes*, *close wardrobe door*. The (iii) transfers ***Situation*** is composed of the following ***Activities***: *lying down*, *sitting down*, *getting up*. Furthermore, concerning the achievement of each one of the aforementioned variables, the activities conforming to such variables must be detected by following a specific sequence: One activity must be finished before the next one can be performed. That is, the precedence criteria must be considered in order for the variable to be completed, as pictured in Figure 1.

After describing the mechanism for orchestrating complex events, the next section describes the proposed operators for representing complex events in the DSL.

#### 4.4.3. Proposed Operators

*Allen’s temporal operators*. In order to deal with the constraints regarding the time dimension, it is necessary to express conditions regarding temporal relations between thesensor states that are relevant for describing activities. To this end, the DSL takes advantage of the criteria over the time dimension mentioned in Section 4.3 and illustrated in Figure 1: concurrency, precedence, simultaneity, and recurrence. For example, the event outlining that shower is “on” during a presence in the bathroom detects an activity described between two sensors. For this purpose, useful tools for representing temporal conditions between two time intervals are the Allen temporal relations [55] (Table 4).

With the aim to establish finite time intervals, temporal bounds relative to Allen’s temporal relations were applied, e.g., the constraint *X MEETS* [t${}_{1}$, t${}_{2}$][0, *∞*) *Y* implies that event *Y* should be *met by* event *X*, that the start time of *X* must occur between $t_{1}$ and $t_{2}$, and that the end time of *X* should occur right before the beginning of *Y*.

**Figure 2:**
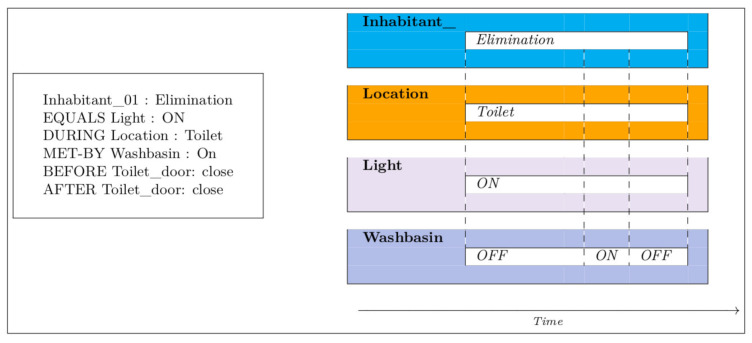
Elimination.

**Figure 3:**
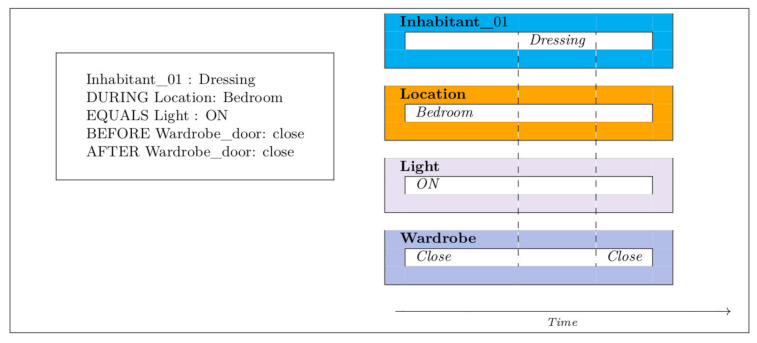
Dressing.

**Figure 4:**
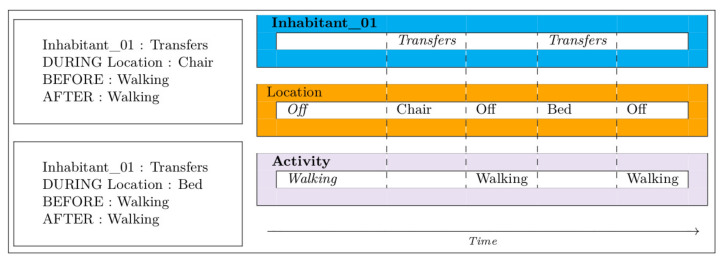
Transfers.

**Figure 5:**
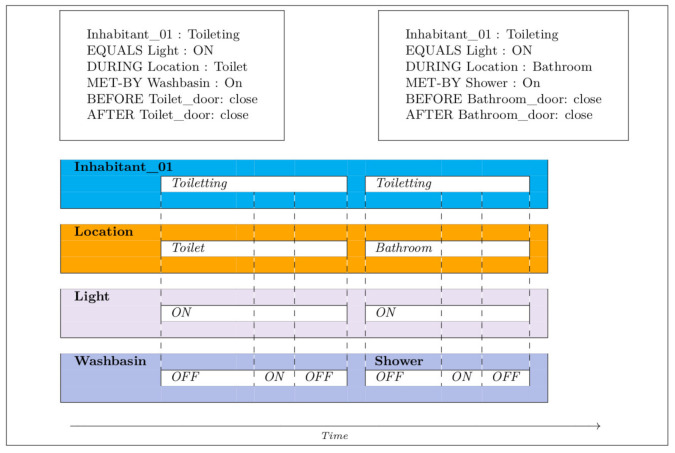
Toileting.

**Figure 6:**
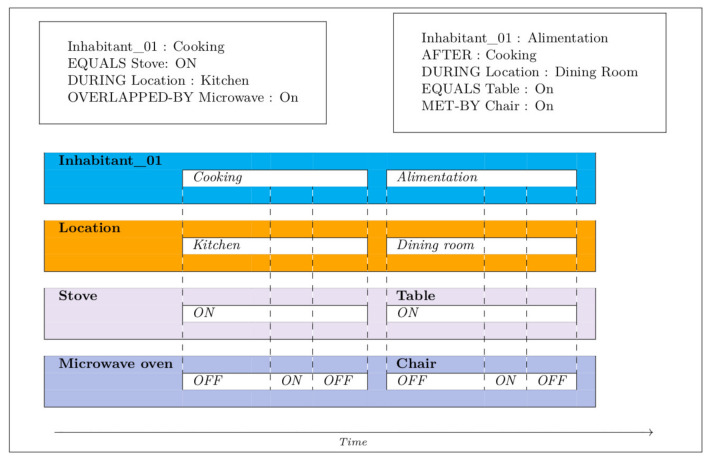
Alimentation.

Figure 2, Figure 3, Figure 4, Figure 5 and Figure 6 present an example of how temporal conditions can be used to model activity recognition in our approach, alongside location based operators. Thus, in order to extend our previous DSL with the identification of complex activities, the operators hereafter are considered.

*Location based operators*. Such operators are in charge of helping to determine the position of the inhabitant within a home environment, as well as the description of ADL through sensor readings. To this effect, the proposed approach considers location based operators, “*Inside*”, “*Outside*”, and “*Joint*”, in order to locate the ADL performed for the inhabitant in space. The descriptions regarding each one of the proposed location based operators are presented in Table 12.

*Event based operators*. Additionally, with regard to the determination of planned tasks that should be carried out by the inhabitant, such as taking medications, event based operators are important for this matter. These operators are presented in Table 13.

The next section is dedicated to the presentation of the framework proposal for the validation and experimentation of the DSL.

## 5. A Framework to Evaluate the AGGIR Variables: Our Approach

Following the proposed DSL, a framework is introduced to fulfill the requirements for processing complex events regarding the AGGIR grid model generated by data recovered from sensors within a smart home environment in order to evaluate the level of independency of elderly people, according to their capabilities in performing activities and interacting with their environments over time.

### 5.1. Framework Modules

The main purpose of the presented framework is to encourage users who are not necessarily acknowledged in the programming field to be able to define events according to the AGGIR grid variables by providing high-level abstraction, which makes it easy and intuitive by means of concepts that are close to the final users.

**Figure 7:**
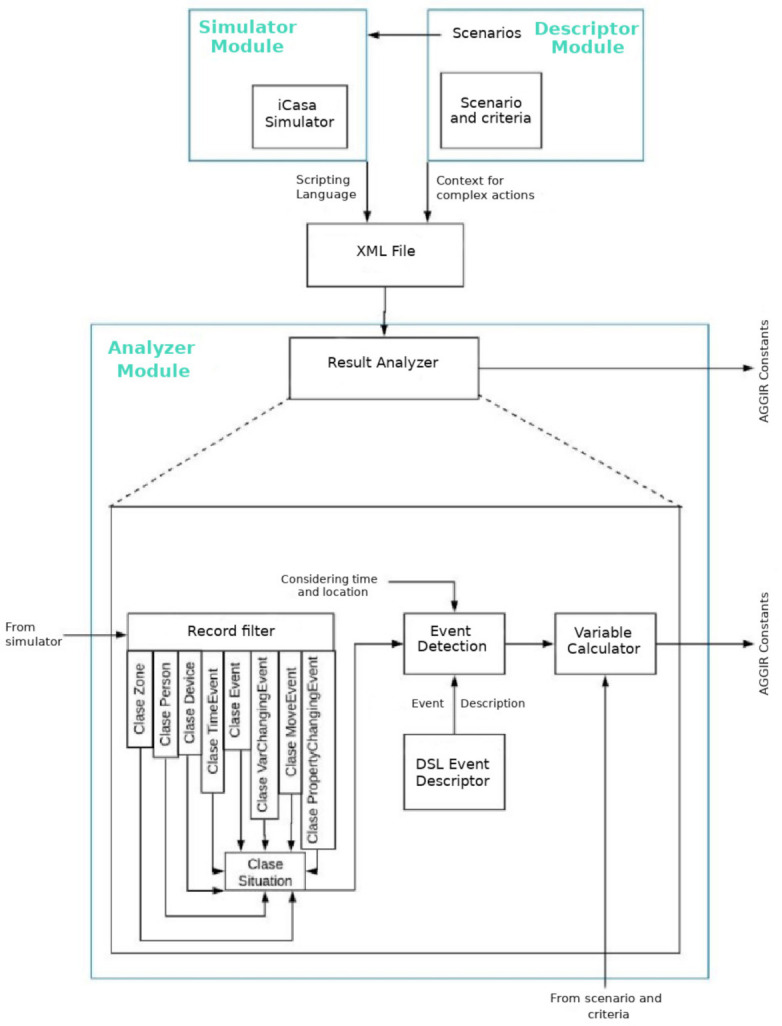
General Architecture of the proposed framework.

The proposed framework is composed by three main modules, as shown in Figure 7: (i) the simulator module; (ii) the descriptor module; and (iii) the analyzer module. All of them are supported by the DSL. A brief description of each one is provided in the next paragraphs.

**Simulator module**: In order to describe the elderly’s ADL, smart-home environment scenarios need to be parameterized. For this purpose, the activities performed by the subject are carried out and information is recovered from sensors located within the smart home environment. As the Simulator module, iCASA [71] was integrated into the proposed framework, which is a smart home simulator that allows control over the following: time, environment, inhabitants, devices, a GUI, scripting facilities (for the environment), and notification facilities [72], as listed in Table 14. iCASA is used in order to set up a simulated scenario.

**Descriptor module**: For the purpose of helping users to interact with the framework, the Descriptor module provides a GUI in order to describe all the criteria over the time dimension, location, and events, as well as the required scenarios for the simulation. Moreover, sensors for identifying the ADL have to be specified through the GUI. A group of sensors that can describe such activities are introduced in Table 7.

Moreover, with regard to the scenario specification concerning a certain period of time, the descriptor module is in charge of the definition of the parameters with respect to location of both ADL and sensors, type of sensors, time, and events that need to be identified: (i) Location Map, which is applied to refer to the representation of the environment by means of the house plan where the implementation of the sensor network takes place; (ii) Sensor network, which is focused on defining information related to the sensor network environment infrastructure, and to this end relevant data should be managed, such as the inventory of sensors located within the smart-home environment, as well as the location where each sensor is implemented; (iii) Sensor reading, due to the fact that the information retrieved from sensors is in raw format, such data need to be organized for the purpose of easing their retrieval and interpretation during the event detection process; (iv) Event condition, since conditions are established for triggering the detection of events, such conditions must be defined by the user; and (v) Event occurrence, where once the event conditions have been provided, the event occurrence must be managed in accordance with such aforementioned conditions.

**Analyzer module:** In order to recover data from the Simulator module and the Descriptor module, the Analyzer module is proposed to carry out such a task. Once all the necessary data are collected, the analyzer module analyzes them in order to classify them and to evaluate if the AGGIR variables of the case study have been carried out to completion. The Analyzer module consists of the following: (i) a record filter; (ii) an event detector; and (iii) a variable calculator. Such components are described hereafter.

*Record Filter*: Once the simulation has been conducted, in order to manage the obtained data, the record filter of the Analyzer module separates the recovered data into a series of records. Each record is responsible for the description of actions collected by the sensor network located within the smart-home environment. Therefore, each register is conformed by data files, i.e., the time when the action takes place (corresponding to the simulator clock), the sensor ID, and the sensed value. Furthermore, because the retrieval of such values is generated in raw data format, it is necessary to translate them by means of the Analyzer module with the aim of achieving the identification of events, as well as calculation of values related to the AGGIR grid variables.

With the aim of performing a filtering of all entries, the first task of the analyzer module is to classify such inputs in a correct manner. For this matter, the generation of “filters” was modeled in the present approach as classes. These classes were introduced in order to determine the type of elements that integrate the analyzed set of instructions by giving each element the appropriate attributes for the modeling of raw data. To this extent, the proposed generated classes for data classification are enlisted in Table 15. Subsequently, in order to detect the performed activities, the *Event detector* is presented.

*Event detector:* After all relevant data from PSN and BSN have been gathered, the identification of events must be carried out. To this extent, the Event detector relies on the introduced DSL to achieve such identification, by managing information grouped in multiple records which are extracted from several sensors.

Aiming to separate each reading detected by several devices, the Event detector retrieves information in terms of the scripting language. Such events are identified as low-level actions instances, e.g., if a presence sensor is off in room X and after a period of time another presence sensor is on in room Y, it can be inferred that the action “walk” was carried out.

Regarding the recognition of malfunctions, the detection of abnormal situations is carried out at the level of identification of events and situations. For this matter, throughout the analysis of low-level events (i.e., Situation class instances) collected from device readings from the simulator module script, it was possible to extract a set of anomalies to be detected by the aforementioned module. Such anomalies are enlisted hereafter in Table 16.

Each one of the aforementioned anomalies is related to the AGGIR grid variables. The detection of any of these issues may return a negative value for the associated variable, which is the inability to satisfactorily carry out the AGGIR variable in question.

*Variable calculator:* Regarding the achievement of the AGGIR variables, the criteria consisting of accomplishing a determined number of events during a specific time lapse related to the evaluated AGGIR variable must be fulfilled, e.g., in order to validate if the dressing AGGIR variable is consummated, the AGGIR dressing event conditions such as moving towards the wardrobe at least twice a day must be met. For this purpose, every AGGIR variable is based on three major states that identify whether the elderly inhabitant possesses the ability to perform the ADL conforming to a specific variable, whether it is conforming completely, partially, or not existent at all. This study covers a proposal which only relies on two cases out of the three above-mentioned options, meaning that either the inhabitant is in complete possession of the skills concerning the performance of the ADL composing the evaluated AGGIR variable or simply not.

### 5.2. Complex Event Detection

Provided the main components of the aforementioned framework proposal, the description of the process for achieving the detection of complex events is then referred.

Due to the fact that ADL takes place within a domestic surroundings, the sensors for detecting such ADL are placed within a smart-home environment. For this matter, such an environment is defined and configured through the Simulator module. Then, the detection process is triggered by the readings obtained from the sensors located within the smart home environment simulator.

In order to obtain information to achieve the identification of the AGGIR variables after every activity has been carried out to completion, some relevant data have to be considered, e.g., *startTime* and *endTime*,as shown in Table 8. All these data are specified in the Descriptor module. After the simulation takes place, a history log file is created, which considers the logical aspect of situations, i.e., if the inhabitant is eating and it is noon, it might seem logical, but the fact that the inhabitant is eating in the toilet is not logical.

As a result, the history log file is necessary for deducting the possible activities that will be performed in the future and to find a relation among them to assure coherent behaviour. Such a file will permit obtaining information on whether it is from long or short periods of time, making possible the identification of complex situations inside the home environment, such as feeding, toileting, and transfers, where data collected through the timeline of activities are useful for determining if the behaviour of the inhabitant can be considered as normal.

## 6. Experimental Evaluation: Use Case Description

**Figure 8:**
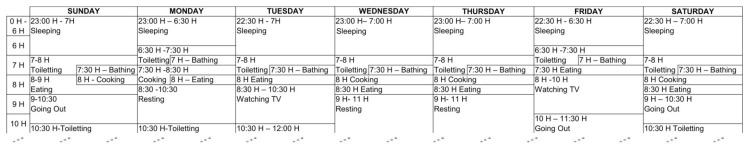
Schedule employed in the simulation.

**Figure 9:**
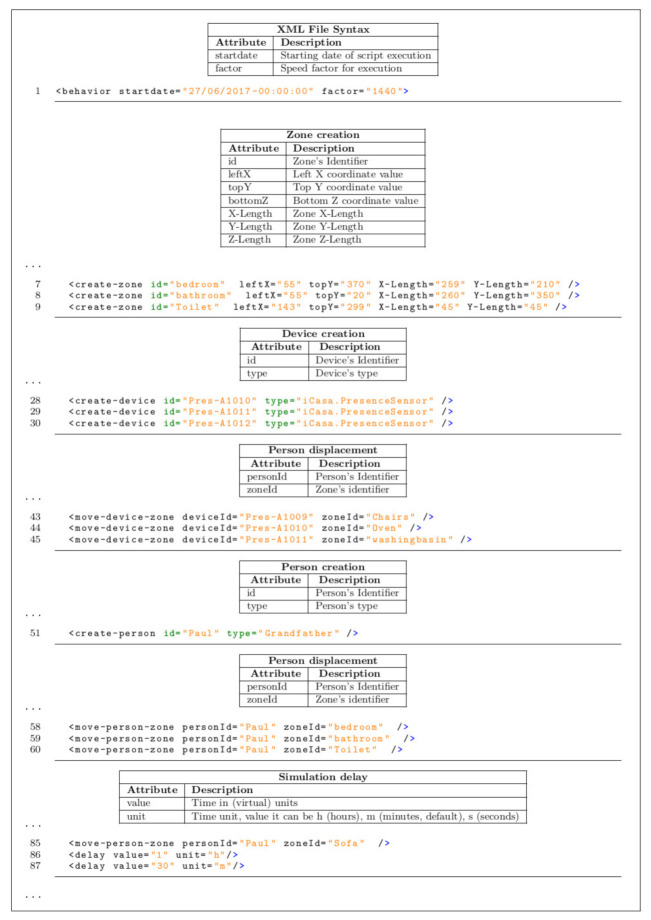
Excerpt of an XML format file scenario employed by the simulator module.

In order to demonstrate the efficacy and suitability of our proposed approach, a set of experiments with regard to the detection of the AGGIR grid variables is performed. For the purpose of evaluating the introduced DSL, four of the AGGIR variables (i.e., dressing, toileting, transfers, and feeding) are picked with the aim of determining their testability in many scenarios by means of records representing the occurrence of ADL performed by the elderly inhabitant within a one week period. In order to detect either the achievement or absence of the AGGIR variables by means of the DSL, we follow a methodological process for the experimental simulations consisting on the following steps, as depicted in Figure 7:

*Step 1*. In order to prepare the scenario for an elderly indoor daily routine over the course of one week, the first step consists of the specification of the criteria over the time and location of activities, type and location of sensors, and the events that have to be detected; in this specific case, all activities related to dressing, toileting, feeding, and transfer AGGIR variables. For this matter, the aforementioned actions are specified and described through the GUI of the Descriptor module. To this end, the primary source of information used to generate the scenario is the schedule proposed by the work of [9] where the behaviour of an inhabitant living in a genuine household environment is simulated based on the daily routine of an elderly resident, as shown in Figure 8. However, in order to render the scenario more suitable for the simulation, many modifications take place. Furthermore, for the purpose of making the scenario more suitable for the simulation, the XML format is used to describe the scenario programmatically, given that such files allow defining events chronologically, thus facilitating the processing carried out by the Simulator module. A brief example of the above-mentioned files is provided in Figure 9.

*Step 2*. Once the information provided by the Descriptor module is established; the Simulator module, i.e., the iCASA framework, executes the simulation according to such precise information.

*Step 3*. After the simulation is performed, the record filter component of the Analyzer module organizes the resulting data into a set of records, each of which represents an action captured by a sensor within the smart home environment (Figure 7). To this end, each record consists of data fields, such as the time it occurred (according to the simulator clock), the sensor ID, and the sensed value. Moreover, due to the fact that such values are raw data, they must be interpreted by the Analyzer module in order to generate the detection of events and then to calculate the values of the AGGIR variables. Subsequently, having collected all the results, the next step is the detection of events. For this purpose, an event is considered as a composite act that is described by data provided by several sensors; that is to say that an event relies on more than one record in raw data. Afterwards, the DSL is in charge of providing an accurate and unified description of the events. Once the event detection has occurred, the aim of the variable calculator within the Analyzer module is to indicate whether an AGGIR variable has been accomplished or not.

In order to test the whole approach, we show its application to several scenarios, as we present in the following section.

### 6.1. Experimental Results

We designed two scenarios for the experimental evaluation. The first scenario consists of an ideal week case scenario in which all the ADLs related to a specific AGGIR variable performed by the inhabitant are conducted successfully. The second scenario is related to the generation of a failure during the daily routine of the inhabitant and randomly drops some of the ADLs conforming a specific AGGIR variable. Moreover, in order to generate the required simulations, the aforementioned scenarios consist of two sets of inputs managed by the proposed framework. Each one of the scenarios is conformed by seven days (one-week period).

In what follows, the main characteristics of the simulation scenarios concerning the identification of ADL as well as the AGGIR grid variables are described. In addition, since evaluating the ability of the proposed approach to deal with larger time domains is required, as well as several records and events, the scalability of the introduced framework is also illustrated.

### 6.2. Toileting, Dressing, and Transfer AGGIR Variables

Conditions regarding the achievement of a certain number of events within a period of time must be accomplished for the evaluation of the AGGIR variables. In order to detect the toileting AGGIR variable, the following conditions must be met: the inhabitant must use the toilet and wash his hands after eating or using the toilet for at least three times a day. To calculate the transfer variable, namely the ability of a person to perform the basic movements of his daily routine such as rising from bed, sitting down, and standing up from a chair, there must be at least three sitting events a day, either taking place in the living room or in the kitchen, and at least one event of rising from the bed one time per day, while the verification of the dressing variable relies on dressing events such as approaching the wardrobe at least twice a day.

In this sense, we represent all these variables as follows. The conditions for the achievement of the toileting AGGIR variable are as follows: (i) the inhabitant must use the toilet; (ii) after, the chain of the toilet flush must be pulled; (iii) after using the toilet, the inhabitant should have his hands washed. In order to analyze such a variable, the records created by the presence sensor located within the bathroom area, alongside the records issued from the use of both the toilet flush sensor and the washbasin proximity sensor, must be examined. To identify the AGGIR dressing variable, the rules for the detection of ADLs composing such a variable are as follows: (i) the inhabitant must be close to the wardrobe area; and (ii) the inhabitant must spend time changing clothes. To this end, data originating from the wardrobe door sensor, as well as the wardrobe proximity sensor, must be gathered. For the purpose of recognizing the AGGIR transfer variable, the following constituent events regarding the aforementioned variable must occur: (i) getting up from bed; (ii) taking a seat; and (iii) standing up from a chair, but not necessarily in this order. In this matter, capacitive sensors located at both the bed and chair/armchair are responsible for the data collection.

**Figure 10:**
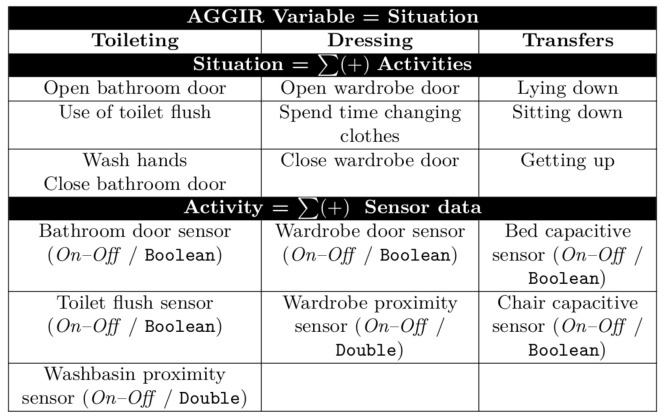
Orchestration of activities/AGGIR Variables.

In order to illustrate the three AGGIR variables outlined above, Figure 10 provides a description of all activities related to the three considered AGGIR variables, as well as sensors related to each one.

With a view to perform the evaluation of the AGGIR variables, all the simulating and processing operations are performed by means of two sets of inputs for the introduced framework in order to generate two different scenarios. For this matter, the week is separated into seven days, each of which is represented by means of one simulation file within the smart home environment simulator.

To this effect, the first scenario is simulated of an ideal week case scenario, meaning that all the ADL were performed with no impediment by the inhabitant throughout the entire week by means of a simulated sensor network within a house environment for monitoring the ADL carried out by the elderly resident. The results obtained from the simulation are presented in Table 17, in which the number of detected activities per day related to each one of the considered AGGIR variables is shown. The check mark means that the criteria for determining that the person is independent (i.e., minimum number of activities related to each variable) has been met.

For the purpose of generating a malfunctioning on the developed criteria, some of the ADL related to the evaluated AGGIR variables were randomly excluded in the second scenario during the seven-day period of analysis. Once the simulation was conducted, the obtained results demonstrate that the detection of complex events regarding either the achievement or the absence of ADL were carried out successfully, according to the pre-established conditions, as listed in Table 18.

**Figure 11:**
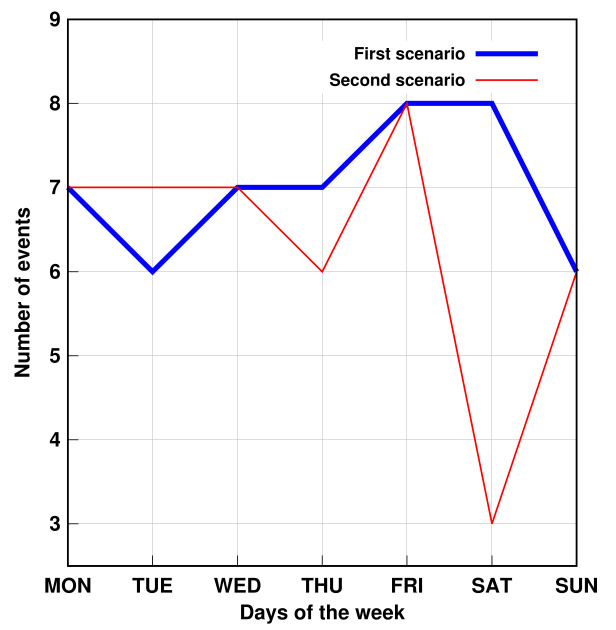
AGGIR Transfer events.

**Figure 12:**
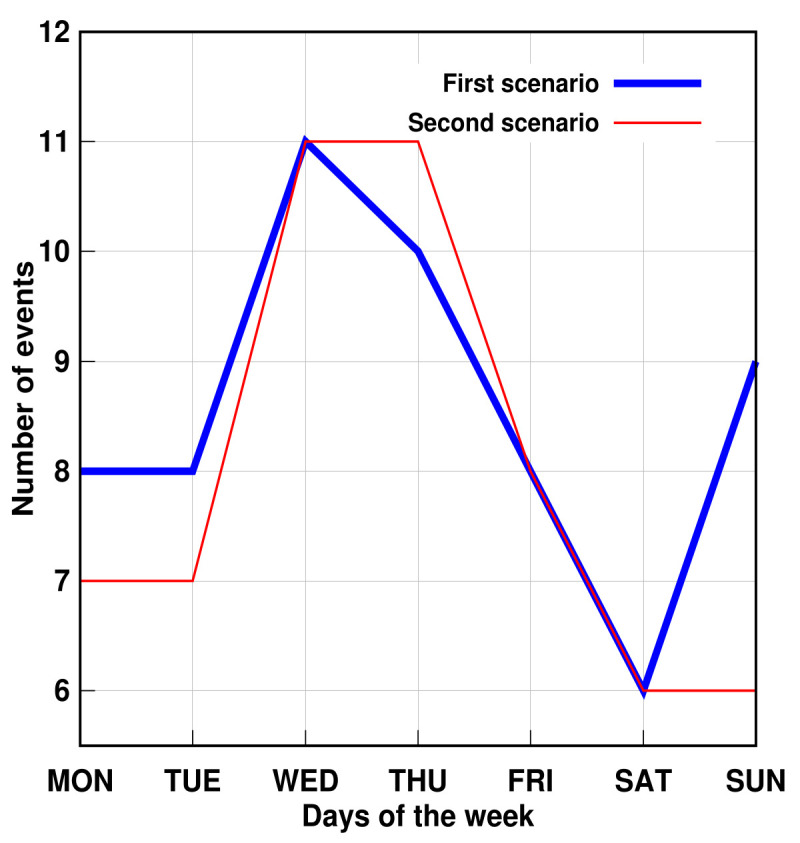
AGGIR Toileting events.

**Figure 13:**
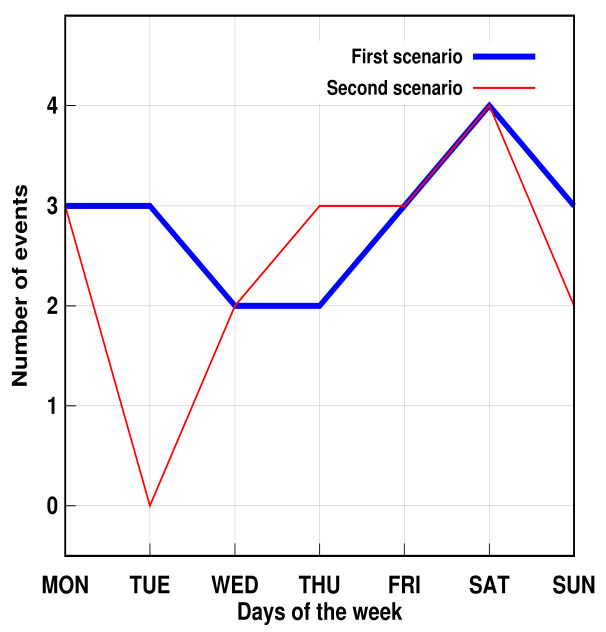
AGGIR Dressing events.

Furthermore, the proposed method succeeded in identifying three simulated problems within a week. The days were randomly chosen. Figure 11 and Figure 12 show that, despite the number of records in the second scenario related to personal transfer and toileting, it did not change significantly compared to the first analyzed scenario. However, a problem with the dressing variable was detected, as illustrated in Figure 13. This, in turn, reflects how the DSL event descriptor can perform a smart analysis of events.

### 6.3. AGGIR Alimentation Variable

With the aim of evaluating the alimentation AGGIR variable, for the first scenario, the ADL related to cooking must be performed by the inhabitant before the alimentation AGGIR variable takes place. That is to say, the accomplishment of preconditioned events conforming to cooking activity is necessary, particularly the following: (i) open/close the refrigerator door; (ii) open/close the kitchen cabinet door; (iii) use of the stove/burners; (iv) use of the traditional/microwave oven and not necessarily in this order. On the other hand, for the second scenario, some of the above-mentioned events concerning the cooking task were dropped. For this matter, information collected from sensors located within the kitchen area must be analyzed. To this end, data originated by both the refrigerator and kitchen cabinet door sensors, the stove magnetic sensor, and the oven/microwave oven sensor must be gathered.

Nonetheless, it is important to remark that according to the AGGIR grid, cooking is not conceived as a discriminatory variable and not as an evaluation criterion for the alimentation AGGIR variable, which only requires the elderly individual to be capable of serving and eating a prepared meal. For this reason, in our approach, cooking is considered as an ADL that can trigger the Alimentation AGGIR variable with the intention of not contradicting the pre-established evaluation criteria, which can also be used for detecting the aforementioned variable in this work.

**Figure 14:**
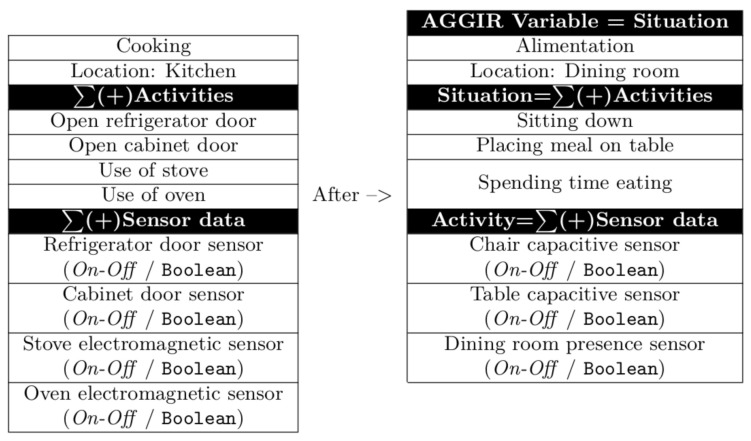
Orchestration of the AGGIR Alimentation Variable.

Once the cooking task is achieved, in addition to the first scenario, events defining the alimentation AGGIR variable must be identified, such as the following: (i) sitting within the dining room area; (ii) placing meal on the table; and (iii) spending time consuming food. Moreover, with the aim of identifying the occurrence of the above-mentioned events, data from sensors located within the dining room area must be collected, such as the following: chair capacitive sensor, table capacitive sensor, and dinning room presence sensor. With respect to the second scenario, some of the ADL conforming to the alimentation AGGIR variable were arbitrarily left out. Additionally, in order to assure a coherent self-feeding behaviour, both scenarios must occur at least three times a day. The orchestration of activities regarding the alimentation AGGIR variable is illustrated in Figure 14.

Having completed the simulations of both scenarios, the results concerning the first scenario proves that, indeed, the detection of complex events conforming the cooking tasks, as well as those from the alimentation AGGIR variable, were carried out to completion successfully and in accordance with the predetermined input conditions, as listed in Table 19 in which check marks indicate that the minimum number of detected events related to the aforementioned variable have been performed satisfactorily.

Moreover, as for the second scenario, due to the fact that some of the activities were not taken into account for the analysis of both the cooking activity and the alimentation AGGIR variable; the proposed framework also succeeded in identifying whether or not the concerned ADL was performed according to initial parameters, as presented in Table 20.

**Figure 15:**
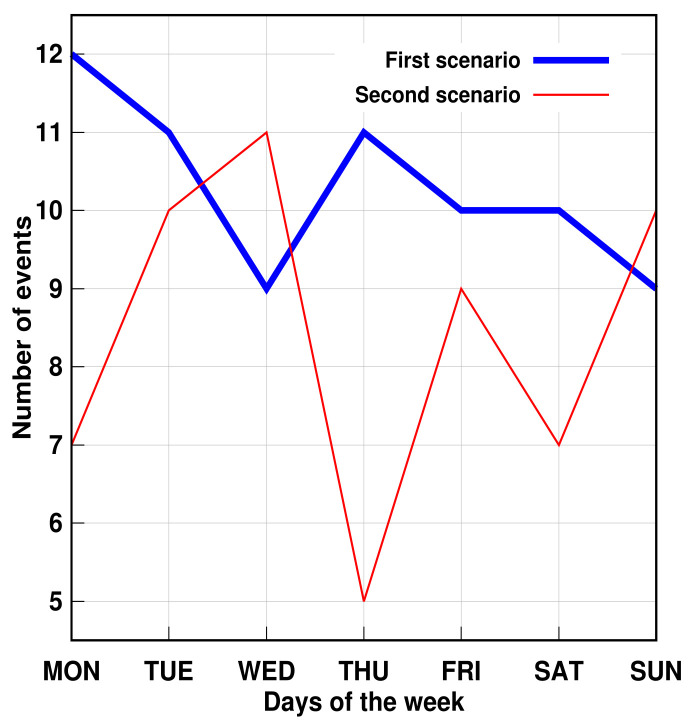
Cooking events.

**Figure 16:**
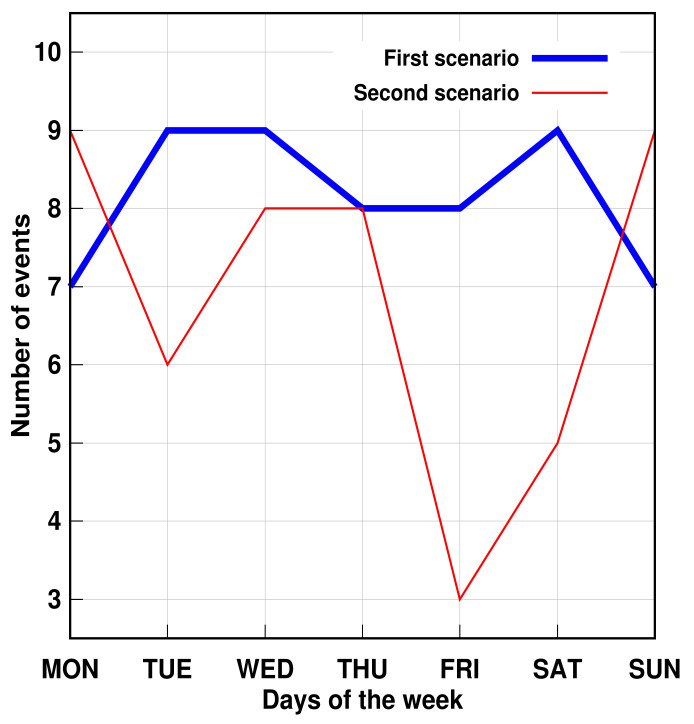
AGGIR Alimentation events.

Furthermore, in order to illustrate the number of events related to the cooking task, as well as for the alimentation AGGIR variable, Figure 15 and Figure 16 introduce the events detected for both scenarios by means of graphical representation. Additionally, it can be observed for each case that the second scenario presents a significant threshold with respect to the first scenario in some of the weekday simulations, which implies that both the cooking activity and the alimentation AGGIR variable were not particularly achieved on those days and, hence, during the evaluated week concerning the second scenario.

### 6.4. Health-Care Device Use Case

In order to prove the flexibility of the proposed framework, a health-care device use case is introduced. This case considers a resident in charge of taking glucose level measurements by means of a glucometer, performing a type of measurement other than those listed in the AGGIR grid. We design two scenarios where, to ensure continuous measuring, it is necessary for the house resident to perform such measurements at least three times a day. To this extent, the first simulation scenario consists of a week during which the person was completely responsible of employing a glucometer device for measuring his own glucose levels, meaning that the completion of a planned medical task was achieved. The second scenario was thought to randomly exclude some of the measurements throughout several days of the week. All the simulations and operations are carried out by the proposed framework by means of records corresponding to each scenario. The results obtained from the simulations corresponding to both scenarios are presented in Table 21.

**Figure 17:**
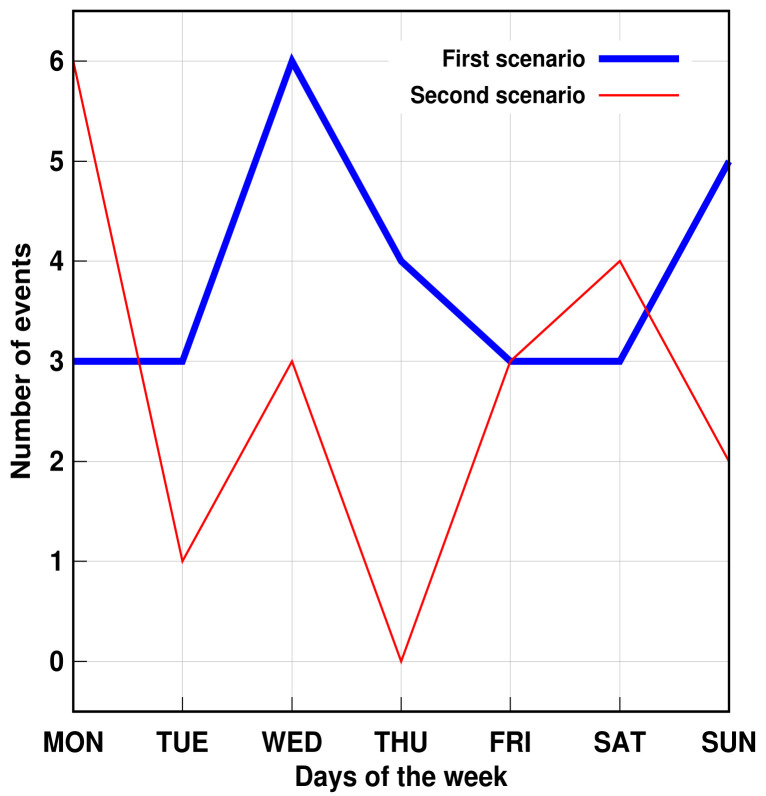
Glucose device events.

With regard to the simulation results, it can be observed in Figure 17 that the events occurring during the simulation week for the first scenario were satisfactorily detected, which implies that the measurements of glucose levels were achieved in accordance with the preset criteria, meaning that the house resident is in charge of planning tasks in a coherent manner. Additionally, information obtained from the second scenario shows that the inhabitant did not succeed in taking charge of a planned task due to the fact that the number of identified events per day was not coherent (three to six times a day) (more precisely, tuesday, thursday, and sunday).

The latter can be interpreted as the efficiency of the framework to perform the recognition of planned tasks concerning measurements carried out by the user by means of medical devices.

We also performed experiments to evaluate the framework scalability and performance In what follows, we describe the results regarding these experiments.

### 6.5. Framework Scalability

**Figure 18:**
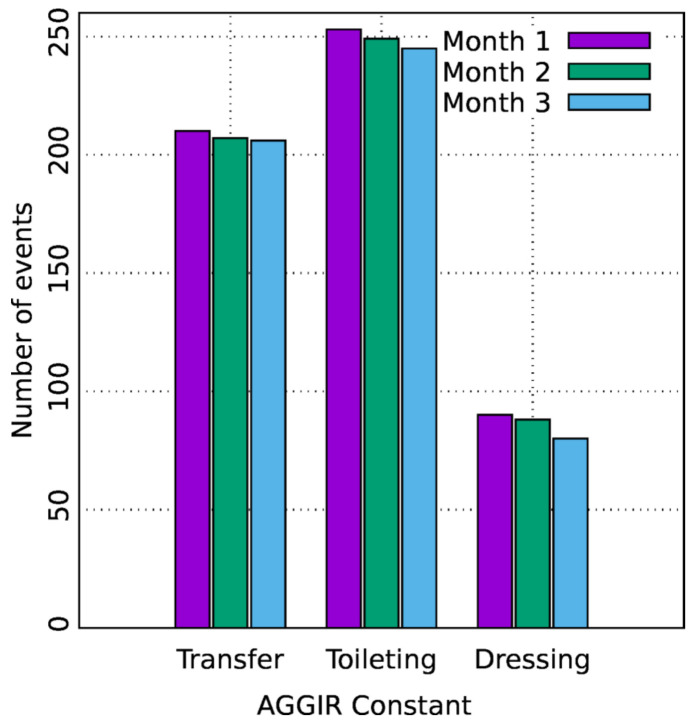
Three-month algorithm scalability.

To validate the execution of the framework under event processing conditions regarding both larger time domains and higher workload cases, the scalability of the framework for a period of time longer than one week was tested. For this matter, a time lapse of three months was simulated in order to generate the corresponding records. Next, the proposed criteria were applied with the intent to create the events and, thus, to calculate the values of the three following AGGIR variables: toileting, transfers, and dressing. In addition, with a view to allow flexibility when processing records and generating events, the assumption that each month is conformed by thirty days was considered. By assuming so, there was a possibility to perform the required simulations by employing an approximate number of days per month, i.e., 28, 29, 30, and 31 days (Figure 18).

Finally, to make sure that the algorithm is scalable, the elapsed time during the calculation of the three above-mentioned AGGIR variables for simulation time periods for one day, one week, two weeks, three weeks, a month, two months, and three months were monitored. Table 22 summarizes the elapsed time for each case. The values displayed include the time spent for each simulation, results analysis, events generation, and finally the calculation of the AGGIR variable.

**Figure 19:**
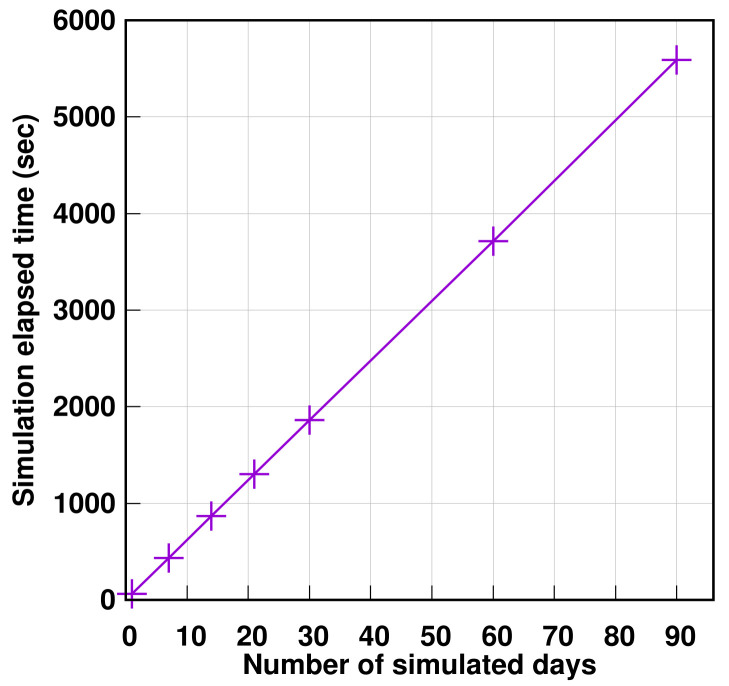
Analysis of Algorithm performance.

In order to present the results thus achieved, Figure 19 shows the performance of the algorithm with simulating scenarios carried out with different time ranges. As a result, the obtained diagram is linear which corresponds to the complexity of the algorithm of the Analyzer module represented by *O*(*n*), where *n* defines the number of events, implying that the framework is scalable as a whole.

With the aim of applying the above-mentioned steps, several use cases are provided, namely the alimentation, toileting, cooking, and transfer AGGIR variables, as well as a description regarding the use case of a medical device for measuring blood glucose level. Moreover, the results obtained show that the proposed framework succeeded on detecting the events; therefore, the AGGIR variables according to pre-established conditions are present in the experimentation scenarios.

## 7. Conclusions

In recent years, it has been reported that the population sector targeted as the elderly is increasing more rapidly than in the previous decades. In addition to that, age-related health issues anddifferent chronic diseases are starting to become apparent at this stage of life. Moreover, various surveys indicate that elderly people are willing to live independently in their own homes for as long as possible. In order to assess the above-mentioned problem, PSNs and BSNs in smart environments represent an option regarding healthcare solutions for monitoring elderly people at home. However, despite their popularity, the absence of a monitoring approach with regard to a dependency evaluation model such as the AGGIR grid variables has never been treated. In this context, the core contributions of this research work are as follows: (i) a state-of-the-art related to the proposed subject and future circumstances with regard to aging population, as well as the capabilities of technology for assisting maintenance and monitoring the health needs of people are reviewed; (ii) a DSL relying on the AGGIR grid evaluation model for assessing the performance of ADLs of the elderly/handicapped at home; (iii) a framework tool for validating the proposed DSL in terms of its capacity for processing complex events; this framework offers a smart-home simulator called iCASA for carrying out evaluation and experimentation and a parser in charge of processing the data issued from the DSL aimed to create the appropriate instructions for the iCASA platform.

We are currently working on proposing the identification of a three-state variable approach for the AGGIR grid. Due to fact that the current version of the introduced DSL is only able to recognise two of the three values (A for complete dependency and C for complete independency) of the AGGIR grid variables, the proposal of an improved version of the DSL comprehending the three states of identification (including B for partial dependency) is one of the possible directions for future work. For the purpose of assessing AGGIR variables that can be considered as more elaborate, such as coherence, further experimentation is required for evaluating crucial activities describing the concerned variable. Even though the presented approach considers the evaluation of the AGGIR discriminatory variables, with the goal of covering the whole spectrum of the AGGIR variables, the integration of the AGGIR illustrative variables is one of the next steps to be taken. Currently, the perspective introduced in this research study relies on the AGGIR grid variables. In order to expand the coverage of the suggested approach, the inclusion of other dependency evaluation models (e.g., the Functional Autonomy Measurement System—SMAF model) represents a field of opportunity for broadening the scope of the present work. In addition to the presence of the house residents, visitors can represent a factor for change regarding household dynamics. For this matter, the adaptability of the proposed approach must be determined by tests concerning unknown users relative to the smart-home environment, as well as the impact that their visit may cause to the actual inhabitants. Despite the fact of having employed a smart-home simulator, as well as a schedule describing the actual activities that were carried out by the user, the application of the present work for real-time use cases is recommended. To this extent, the monitoring of ADL within an actual smart-home environment would be ideal for testing the present approach in a real-time scenario.

## Figures and Tables

**Table 1:** AGGIR variables.

Discriminatory	Illustrative Variables
Coherence	Management
Location	Cooking
Toileting	Housekeeping
Dressing	Transportation
Self-feeding/Alimentation	Purchases
Elimination	Medical treatment
Transfers	Leisure activities
Indoor movement	
Outdoor movement	
Distant communication	

**Table 2:** Categories of DSL design guidelines proposed by Karsai et al. [29].

Category	Description	Guidelines
Language purpose	Goal of the DSL	1. Identify language uses early.
		2. Ask questions.
		3. Make your language consistent.
Language realization	Implementation of the DSL	4. Decide carefully whether to use graphical or textual realization.
		5. Compose existing languages where possible.
		6. Reuse existing language definitions.
		7. Reuse existing type systems.
Language content	Elements of the DSL	8. Reflect only the necessary domain concepts.
		9. Keep it simple.
		10. Avoid unnecessary generality.
		11. Limit the number of language elements.
		12. Avoid conceptual redundancy.
		13. Avoid inefficient language elements.
Concrete syntax	Readable (external) representation of the DSL	14. Adopt existing notations domain experts use.
		15. Use descriptive notations.
		16. Make elements distinguishable.
		17. Use syntactic sugar appropriately.
		18. Permit comments.
		19. Provide organizational structures for models.
		20. Balance compactness and comprehensibility.
		21. Use the same style everywhere.
		22. Identify usage conventions.
Abstract Syntax	Internal representation of the DSL	23. Align abstract and concrete syntax.
		24. Prefer layout which does not affect translation from concrete to abstract syntax.
		25. Enable modularity.
		26. Introduce interfaces.

**Table 3:** Comparison among the most recent DSL approaches regarding ADLs.

Work	Services	Activities	Sensing	Sensing Type	Geriatric Models
[45]	Movement tracking	IADL	Single	BSN	No
[43]	Activity-oriented context-aware applications, energy	ADL	Multi	PSN	No
[44]	Wellness determination, fall detection, movement tracking, energy	IADL	Multi	PSN BSN	No
[42]	Detection of elderly behaviour patterns, movement tracking	ADL, IADL	Multi	PSN BSN	No
[46]	ADL estimation, energy	ADL	Multi	PSN	No
[47]	ADL estimation, energy	ADL	Multi	PSN	No
[48]	ADL estimation, energy	ADL	Multi	PSN	No
[49]	Assisting meal preparation	IADL	Multi	PSN	No

**Table 4:** Allen’s temporal operators.

Relation	Symbol	Symbol for Inverse	Illustration
X before Y	<	> (after)	*__ X__*
			*__ Y__*
X equals Y	=	= (equals)	*__X__*
			*__Y__*
X meets Y	m	mi (met by)	*___X___*
			*___Y___*
X overlaps Y	o	oi (overlapped by)	*____X____*
			*____Y____*
X during Y	s	di (contains)	*__X__*
			*_____Y_____*
X starts Y	d	si (started by)	*___X___*
			*_____Y_____*
X finishes Y	f	fi (finished by)	*___X___*
			*______Y______*

**Table 5:** Shanahan’s event calculus formulas.

Formula	Meaning
Initiates (α, β, τ)	Fluent β starts to hold after action α at time τ
Terminates (α, β, τ)	Fluent β ceases to hold after action α at time τ
InitiallyP (β):	Fluent β holds from time 0
$\tau_{1}$≤$\tau_{2}$	Time point $\tau_{1}$ is before time point $\tau_{2}$
Happens (α, τ)	Action α occurs at time τ
HoldsAt (β, τ)	Fluent β holds at time τ
Clipped ($\tau_{1}$, β, $\tau_{2}$)	Fluent β is terminated between times $\tau_{1}$ and $\tau_{2}$

**Table 6:** Spatial, temporal and event operators.

	Temporal	Location	Event
Freksa [57]	☑	□	□
Ribarić and Dalbelo Bašić [58]	☑	□	□
Randell et al. [59]	□	☑	□
Galton et al. [60]	□	☑	□
Randell et al. [61]	□	☑	□
Kim et al. [62]	□	☑	□
Pecora et al. [63]	□	☑	□
Bruno et al. [64]	□	☑	□
Patkos et al. [65]	□	☑	□
Santos et al. [66]	□	☑	□
Furze and Bennett [67]	□	☑	☑
Angsuchotmete et al. [68]	□	☑	☑

**Table 7:** Smart Home Sensors.

Sensor Type	Attributes	Data Type	Examples
Electromagnetic sensor	On/Off	Boolean	Cooker/stove; oven; light; switch
Proximity sensor	On/Off	Double	Sink
Capacitive sensor	On/Off	Boolean	Kitchen; counter; chair
Magnetic sensor	Open/Close	Boolean	Refrigerator door; cupboard doors
Presence Sensor	On/Off	Boolean	Room occupancy

**Table 8:** Additional data for recognition of activities.

Extra Data Type for Each Task	Data Type
startTime	Date/String
endTime	Date/String
Duration	Integer/Double
Location	String
Day	String/Integer

**Table 9:** Medical sensors [69].

Device	Parameters	Units	Collected Data	
			Data Type	Range
Pulse and Oxygen in Blood (SPO2)	Pulse: 25–250 SPO2: 35–100	PPM (peaks per minute) %	Integer Integer	25–250 PPM 35–100%
Electrocardiogram (ECG)	Heart rate ECG signal	BPM (beats per minute) Volts	Integer Real	0–200 BPM 0/5 V
Airflow	Respiratory rate Breathing intensity	PPM Volts	Integer Real	0–60 PPM 0–3.3 V
Blood Pressure Sensor	Systolic pressure Diastolic pressure Pulse	mm Hg mm Hg PPM	Integer Integer Integer	0–300 mmHg 0–300 mmHg 30~200 PPM
Glucometer	Glucose Glucose	mmol/L mg/dL	Real Real	
Body Temperature Sensor (BTS)	Body Temperature	Degree Celsius (°C)	Real	0–50 °C
Electromyography (EMG)	Muscle rate Muscle signal	CPM (contractions per minute) Volts	Integer Real	0–60 CPM 0–5 V
Spirometer (for breathing measuring)	Volume Air flow	L L/min	Real Integer	0.01~9.99 L 50~900 L/min
Galvanic skin response (GSR)	Conductance Resistance Voltage	Siemens Ohms Volts	Real Real Real	0–20 Siemens 10–100 K Ohms 0–5 V
Body Position	Body position Acceleration	Human body position G	String Real	5 positions
Snore	Snore rate Snore signal	SPM (Snores per minute) Volts	Integer Real	0–60 SPM 0–5 V

**Table 10:** Orchestration of activities.

Elimination	Dressing	Transfers
Activity = ∑(+) Sensor data		
Bathroom door sensor	Wardrobe door sensor	Bed capacitive
(*On–Off*/ Boolean)	(*On–Off*/Boolean)	sensor (*On–Off*/Boolean)
Toilet flush sensor	Wardrobe proximity	Chair capacitive
(*On–Off*/Boolean)	sensor (*On–Off*/Double)	sensor (*On–Off*/Boolean)
Washbasin proximity		
sensor (*On–Off*/Double)		

**Table 11:** Orchestration of the AGGIR variables.

AGGIR Variable = Situation		
Elimination	Dressing	Transfers
Situation = ∑(+) Activities		
Open bathroom door	Open wardrobe door	Lying down
Use of toilet flush	Spend time changing clothes	Sitting down
Wash hands Close bathroom door	Close wardrobe door	Getting up

**Table 12:** Location-based operators.

Operator	Description	Data Type
*Inside*	Returns true if the user is inside a given area.	String
*Outside*	Returns true if the user is outside a given area.	String
*Joint*	Returns true if the user is in two locations at the same time.	String

**Table 13:** Event-based operators.

Operator	Description	Data Type
*Planned task (medical* *measurements)*	Returns true if the user performs a planned task.	String
*Unplanned tasks (medical* *measurements)*	Returns true if the user performs an unplanned task.	String

**Table 14:** iCASA facilities.

Time	Possibility to slow down, speed up, or stop time during the simulation. Simulation of long-term actions such as energy consumption to skip to important actions.
Environment	Definition of different zones in a house. An administration interface to modify different physical properties (temperature, luminosity, etc.) of the different zones is provided.
Inhabitants	Insertion or removal inhabitants from the environment. Inhabitants can move from zone to zone and may be carrying physical devices.
Devices	Devices can be simulated or real. At any time, the user can add or remove new simulated devices and modify their localisation in the rooms.
A graphical user interface	The interface displays a map of the house and the localisation of the different devices. It permits creating and configuring devices and can create and move physical users and watch their actual configurations.
Scripting facilities	Support relative to the scripts written to control the environment. Scripts provide a convenient method for testing the applications under reproducible conditions.
Notifications facilities	iCASA is event-based and is able to notify subscribers of any modifications in the environment.

**Table 15:** Classes for data classification.

Class Name	Function
***Zone***	Modelling the simulator zones; in charge of identifiers, measurements, variables, and their values.
***Device***	Managing several aspects concerning the simulator devices, i.e., identifiers, properties, and values spatial location.
***Person***	Controlling the inhabitants within the smart-home environment, such as the person identifiers and their location with respect to the simulation context.
***TimeEvent***	Modelling the instructions concerning the time dimension: generating real time data parting from time raw data, allowing time management during the analysis.
***Event***	Considered as a basic event; modelling situations not requiring additional data.
***MoveEvent***	Modelling events regarding movement within a specific zone in the smart-home area, either a person or a device. Subclass of the *Event* class.
***VarChangingEvent***	Modelling events concerning modifications to designated variables to a specific zone, i.e. temperature change. Subclass of the *Event* class.
***PropertyChangingEvent***	Modelling events that generate modifications with regard to the intrinsic properties with respect to the simulation devices.
***Situation***	Modelling complex situations. Complex situations are a result of the interaction of several atomic events that allows identifying abnormal behaviour

**Table 16:** Anomalies identified by the event detector module.

Sensor/Activity	Detected Problem	Issue for the Inhabitant
FloodSensor	Leak or break in pipelines	Flood within the home environment.
DimmerLight/	Exceeded MAX time ON	High energy cost; neglecting of ADLs; location and coherence.
BinaryLight	ON at a wrong time	
Heater/Cooler	Exceeded MAX time ON	High cost, neglect; abnormal thermal sensation; location problem.
	Is ON when not needed	Neglecting household tasks, location, and coherence.
Not leaving room	Exceeded MAX time ON	Housekeeping, alimentation, elimination, and leisure activities.
Light/Presence	Inhabitant stays still	Accident in ZONE, location, coherent behaviour, and transfer.
*CO*/*CO${}_{2}$* sensor	High *CO*/*CO${}_{2}$* concentration	Household tasks, i.e., impossibility to check on the status on pipelines
Door sensor	Door *LEFT OPEN*	Location, coherence, household maintaining.
Siren	Resident must leave the house	Maintenance of home.
Toilet sensors: Door toilet flush, washbasin	Irregular urination time	Toileting, elimination, personal hygiene, and transfers.
Wardrobe door	Not changing clothes	Dressing, coherence, and toileting.
Motion sensor	Wandering around at unusual time	Location and coherence.
Temperature/Motion	Abandoning kitchen while cooking	Location, coherence, and household tasks.

**Table 17:** Results of the first scenario.

		Transfer	Toileting	Dressing
**No. of events**	**MON**	☑ 7	☑ 8	☑ 3
	**TUE**	☑ 6	☑ 8	☑ 3
	**WED**	☑ 7	☑ 11	☑ 2
	**THU**	☑ 7	☑ 10	☑ 2
	**FRI**	☑ 8	☑ 8	☑ 3
	**SAT**	☑ 8	☑ 6	☑ 4
	**SUN**	☑ 6	☑ 9	☑ 3
	**Total**	☑ 49	☑ 59	☑ 19

**Table 18:** Results of the second scenario.

		Transfer	Toileting	Dressing
**No. of events**	**MON**	☑ 7	☑ 7	☑ 3
	**TUE**	☑ 7	☑ 7	☒ 0
	**WED**	☑ 7	☑ 11	☑ 2
	**THU**	☑ 6	☑ 11	☑ 3
	**FRI**	☑ 8	☑ 8	☑ 3
	**SAT**	☒ 3	☑ 6	☑ 4
	**SUN**	☑ 6	☒ 6	☑ 2
	**Total**	☑ 44	☒ 56	☑ 16

**Table 19:** Results of the first scenario.

		Cooking	Alimentation
**No. of events**	**MON**	☑ 12	☑ 7
	**TUE**	☑ 11	☑ 9
	**WED**	☑ 9	☑ 9
	**THU**	☑ 11	☑ 8
	**FRI**	☑ 10	☑ 8
	**SAT**	☑ 10	☑ 9
	**SUN**	☑ 9	☑ 7
	**Total**	☑ 72	☑ 57

**Table 20:** Results of the second scenario.

		Cooking	Alimentation
**No. of events**	**MON**	☒ 7	☑ 9
	**TUE**	☑ 10	☒ 6
	**WED**	☑ 11	☑ 8
	**THU**	☒ 5	☑ 8
	**FRI**	☑ 9	☒ 3
	**SAT**	☒ 7	☒ 5
	**SUN**	☑ 10	☑ 9
	**Total**	☒ 59	☒ 48

**Table 21:** Results of the first scenario.

		First Scenario	Second Scenario
**No. of events**	**MON**	☑ 3	☑ 6
	**TUE**	☑ 3	☒ 1
	**WED**	☑ 6	☑ 3
	**THU**	☑ 4	☒ 0
	**FRI**	☑ 3	☑ 3
	**SAT**	☑ 3	☑ 4
	**SUN**	☑ 5	☒ 2
	**Total**	☑ 27	☒ 19

**Table 22:** Real time elapsed for calculating the constants in seven different simulation time ranges (***D***ay, ***W***eek, and ***M***onth).

Time Period	1*D*	1*W*	2*W*	3*W*	1*M*	2*M*	3*M*
**No. of days**	1	7	14	21	30	60	90
**Time (s)**	62	434	867	1301	1863	3713	5590

## Abbreviations

The following abbreviations are used in this manuscript:

ADL	Activities of Daily Living;
AGGIR	Autonomy Gerontology Iso-Resources Groups;
AI	Artifitial Intelligence;
BPM	Beats per minute;
BSN	Body Sensor Networks;
BTS	Body Temperature Sensor;
CPM	Contractions per minute;
DSL	Domain Specific Language;
ECG	Electrocardiogram;
EMG	Electromyography;
GSR	Galvanic skin response;
GUI	Graphical User Interface;
IoT	Internet of things;
PPM	Peaks per minute;
PSN	Personal Sensor Networks;
SMAF	Functional Autonomy Measurement System;
SPM	Snores per minute.
